# Atrophy of hippocampal subfields relates to memory decline during the pathological progression of Alzheimer’s disease

**DOI:** 10.3389/fnagi.2023.1287122

**Published:** 2023-12-12

**Authors:** Yaqiong Xiao, Yubin Hu, Kaiyu Huang, the Alzheimer’s Disease Neuroimaging Initiative

**Affiliations:** Center for Language and Brain, Shenzhen Institute of Neuroscience, Shenzhen, China

**Keywords:** Alzheimer’s disease, mild cognitive impairment, structural MRI, hippocampal subfields, cognitive decline, early detection

## Abstract

**Background:**

It has been well documented that atrophy of hippocampus and hippocampal subfields is closely linked to cognitive decline in normal aging and patients with mild cognitive impairment (MCI) and Alzheimer’s disease (AD). However, evidence is still sparce regarding the atrophy of hippocampus and hippocampal subfields in normal aging adults who later developed MCI or AD.

**Objective:**

To examine whether atrophy of hippocampus and hippocampal subfields has occurred in normal aging before a diagnosis of MCI or AD.

**Methods:**

We analyzed structural magnetic resonance imaging (MRI) data of cognitively normal (CN, *n* = 144), MCI (*n* = 90), and AD (*n* = 145) participants obtained from the Alzheimer’s Disease Neuroimaging Initiative. The CN participants were categorized into early dementia converters (CN-C) and non-converters (CN-NC) based on their scores of clinical dementia rating after an average of 36.2 months (range: 6–105 months). We extracted the whole hippocampus and hippocampal subfields for each participant using FreeSurfer, and analyzed the differences in volumes of hippocampus and hippocampal subfields between groups. We then examined the associations between volume of hippocampal subfields and delayed recall scores in each group separately.

**Results:**

Hippocampus and most of the hippocampal subfields demonstrated significant atrophy during the progression of AD. The CN-C and CN-NC groups differed in the left hippocampus–amygdala transition area (HATA). Furthermore, the volume of presubiculum was significantly correlated with delayed recall scores in the CN-NC and AD groups, but not in the CN-C and MCI groups.

**Conclusion:**

Hippocampal subfield atrophy (i.e., left HATA) had occurred in cognitively normal elderly individuals before clinical symptoms were recognized. Significant associations of presubiculum with delayed recall scores in the CN-NC and AD groups highlight the essential role of the hippocampal subfields in both early dementia detection and AD progression.

## Introduction

Accumulating literature has suggested that atrophy of hippocampus is closely linked to cognitive decline in normal aging and the progression of Alzheimer’s disease (AD) (Hampel et al., 2008; Shi et al., 2009; Frisoni et al., 2010; Mu and Gage, 2011; Xiao et al., 2023). Indeed, the hippocampus, known to play a crucial role in the formation, organization, and consolidation of new memories, is one of the earliest structures that are vulnerable to atrophy in normal aging, and it also has been acknowledged as the core biomarker for the pathological changes of mild cognitive impairment (MCI) and AD (Mu and Gage, 2011).

While most of neuroimaging studies examined the hippocampus as a single unitary entity, it is recognized that the cytoarchitecture of hippocampus is not homogeneous. The hippocampus can be divided into several anatomically and functionally diverse subfields, which have different connectivity to other brain regions and different vulnerability to disease (Freund and Buzsáki, 1996; Small et al., 2011; Aggleton, 2012; Maruszak and Thuret, 2014). In recent years, with the emergence of high-resolution MRI data acquisition and the development of segmentation techniques (Iglesias et al., 2015), a growing number of studies (Maruszak and Thuret, 2014; de Flores et al., 2015; Zheng et al., 2018; Zhao et al., 2019; Huang et al., 2020) examined the atrophy of hippocampal subfields instead of the hippocampus as a whole. The analysis of hippocampal subfields can better detect subtle changes in the hippocampal structure, more precisely discriminate cognitive correlates of these changes, and identify participants at higher risk of cognitive decline (Csernansky et al., 2005; Carlesimo et al., 2015; de Flores et al., 2015).

A number of studies have investigated the atrophy of hippocampal subfields related to MCI and AD (Mueller et al., 2007; Carlesimo et al., 2015; Wang et al., 2018; Zheng et al., 2018; Krajcovicova et al., 2019; Zhao et al., 2019; Huang et al., 2020). An early study by Mueller et al. (2007) demonstrated reduced volume of CA1 and subiculum in AD patients as compared to age-matched controls. In a study with MCI, AD, and healthy control participants, the authors found the volume of presubiculum and subiculum presented the most remarkable reduction in MCI and AD patients, and in MCI patients, volume of presubiculum and subiculum predicted individual’s performance on the memory tests (Carlesimo et al., 2015). Kälin et al. (2017) found reduced volumes of all bilateral hippocampal subfields except for CA2-CA3 in MCI patients who later converted to AD, and reduced volumes of all bilateral hippocampal subfields at the time of conversion to AD. The study by Zhao et al. (2019) reported significant changes in most of the hippocampal subfields at different stages of AD, and volume of left subiculum was significantly correlated with memory performance. Research has also reported alternations in structural covariance of the hippocampal subfields in subcortical vascular MCI and amnestic MCI patients as compared to cognitively normal (CN) participants, and also found differences in structural covariance of the hippocampal subfields between these two types of MCI patients (Wang et al., 2018). Together, these findings suggest that the analysis of hippocampal subfields may serve as a promising approach to better understand the pathological progression of AD.

A few studies have examined the changes of hippocampal subfields in the CN individuals who were later converted to early dementia (Csernansky et al., 2005; Apostolova et al., 2010; de Flores et al., 2015; Hsu et al., 2015). Csernansky et al. (2005) followed 49 CN subjects with clinical dementia rating (CDR) score of 0 for an average of 5 years, and the authors reported progressive atrophy of left CA1 in subjects who later had a CDR score of 0.5 as compared to those remained a CDR score of 0. The study by Apostolova et al. (2010) analyzed the longitudinal changes of hippocampal subfields in CN subjects and showed greater CA1 and subiculum atrophy in CN individuals who later developed MCI or AD. Hsu et al. (2015) found significantly greater hippocampal tail, presubiculum, subiculum, and total hippocampal atrophy in CN elderly participants who had amyloid-β accumulation compared to those who had no amyloid-β accumulation. These reports suggest hippocampal subfield measurements may be sensitive markers of AD progression and potential biomarkers for early MCI or AD detection. However, little is known regarding the hippocampal subfield atrophy in those who had normal cognition but developed dementia (MCI or AD) later.

In this study, we investigated the atrophy of hippocampus and hippocampal subfields in normal aging adults who had a CDR score of 0 at the time of MRI scan and later converted to a CDR score of 0.5, and MCI and AD patients. Specifically, we obtained a cohort of CN, MCI, and AD participants from the Alzheimer’s Disease Neuroimaging Initiative (ADNI) and categorized CN participants into early dementia converters (CN-C) and non-converters (CN-NC) based on their later CDR score. The comparisons of hippocampal subfield volumes were conducted between groups. We expected significant differences in hippocampal subfield volumes between CN, MCI, and AD groups. We also expected group differences between CN-C and CN-NC participants as neuroanatomical abnormalities of the hippocampus may predict future onset of dementia in cognitively normal elders (Csernansky et al., 2000, 2005; Apostolova et al., 2010). Further, we examined the associations of hippocampal subfields with delayed recall scores (i.e., memory index scores, MIS) as measured by the Montreal Cognitive Assessment (MoCA), a commonly used screening tool for cognitive impairment (Nasreddine et al., 2005). The associations were conducted using regression analyses for the CN-NC, CN-C, MCI, and AD groups, separately. We expected significant relationships between volumes of hippocampal subfields and delayed recall scores as such relationships have been consistently reported in previous studies (Wang et al., 2018; Zheng et al., 2018; Zhao et al., 2019).

## Materials and methods

### Participants

The data included in the present study were obtained from the ADNI database.^1^ The ADNI was launched in 2003 as a public-private partnership, led by Principal Investigator Michael W. Weiner, MD. The primary goal of ADNI has been to test whether serial MRI, positron emission tomography (PET), other biological markers, and clinical and neuropsychological assessment can be combined to measure the progression of MCI and early AD. For more details about this database, please refer to the website^2^ and previous publications (Weiner et al., 2010, 2017; Aisen et al., 2015).

Specifically, a total of 379 participants were obtained from the ADNI database, including 144 CN (66 M/78 F; mean age = 74.78 ± 7.79 years), 90 MCI (48 M/42 F; mean age = 72.56 ± 9.16 years), and 145 AD (84 M/61 F; mean age = 74.94 ± 7.5 years); participants in the CN, MCI, and AD groups were matched on age and gender (*p*s > 0.05). All the participants had demographic information (i.e., age, gender) and scores of CDR and the Mini Mental State Examination (MMSE). Years of education (CN: *n* = 138; MCI: *n* = 89; AD: *n* = 138) and the MoCA (CN: *n* = 134; MCI: *n* = 79; AD: *n* = 84) were collected from most of the participants.

In the analysis, the nearest available CDR assessment before or after the selected MRI scan was used as the baseline assessment, and subsequent CDR assessments were also collected for each participant. Following previous studies (Marquis et al., 2002; Csernansky et al., 2005), we categorized the CN participants into early dementia converters (i.e., CN-C) and non-converters (i.e., CN-NC), where CN-C were defined as those who had a change of CDR score from 0 to 0.5 and CN-NC were those who remained a CDR score of 0. Among 144 CN participants, 116 were identified as CN-NC (53 M/63 F; mean age = 73.98 ± 7.82 years) and 28 were identified as CN-C (13 M/15 F; mean age = 78.1 ± 6.8 years). The CN-C participants had a CDR score of 0.5 after an average of 36.2 months (range: 6–105 months). Table 1 summaries the demographic and clinical details of participants in this study.

**Table 1:** Demographic and clinical information of CN-NC, CN-C, MCI, and AD groups.

	CN-NC (*n* = 116)		CN-C (*n* = 28)		MCI (*n* = 90)		AD (*n* = 145)	
	Mean ± SD	Range	Mean ± SD	Range	Mean ± SD	Range	Mean ± SD	Range
Sex (M/F)	53/63		13/15		48/42		84/61	
Age (years)	73.98 ± 7.82	58.4–91.4	78.1 ± 6.8	66.7–93.2	72.56 ± 9.16	55.2–97.4	74.94 ± 7.5	56–91
Education (years)	17.02 ± 2.31	12–20	16 ± 2.09	12–20	16.08 ± 2.67	8–20	15.52 ± 2.66	8–20
CDR	0 ± 0	0–0	0 ± 0	0–0	0.52 ± 0.09	0.5–1	0.81 ± 0.35	0.5–2
MMSE	29.03 ± 1.08	26–30	29 ± 1.19	25–30	27.27 ± 2.43	16–30	22.72 ± 3.04	5–29
MoCA Total	24.36 ± 1.77	18–28	24.18 ± 1.89	20–27	22.63 ± 3.47	10–29	18.14 ± 4.75	9–27
MoCA MIS	7.55 ± 2.57	0–13	7.46 ± 3.25	0–12	7.68 ± 3.58	0–14	6.88 ± 3.72	0–15

CN-NC, CN non-converters; CN-C, CN converters; MCI, mild cognitive impairment; AD, Alzheimer’s disease; CDR, Clinical Dementia Rating; MMSE, Mini Mental State Examination; MoCA, Montreal Cognitive Assessment; MIS, Memory Index Score.

### MRI data collection

T1-weighted structural MRI brain scans of all 379 participants were used in this study. For detailed information regarding ADNI’s image acquisition protocols, which are different for multiple MRI scanner types used in ADNI, see http://adni.loni.usc.edu/methods/documents/mri-protocols/. Raw Digital Imaging and Communications in Medicine (DICOM) MRI scans were downloaded from the public ADNI site,^3^ reviewed for quality, and automatically corrected for spatial distortion caused by gradient nonlinearity and B1 field inhomogeneity.

### MRI data processing and hippocampal subfield segmentation

**Figure 1:**
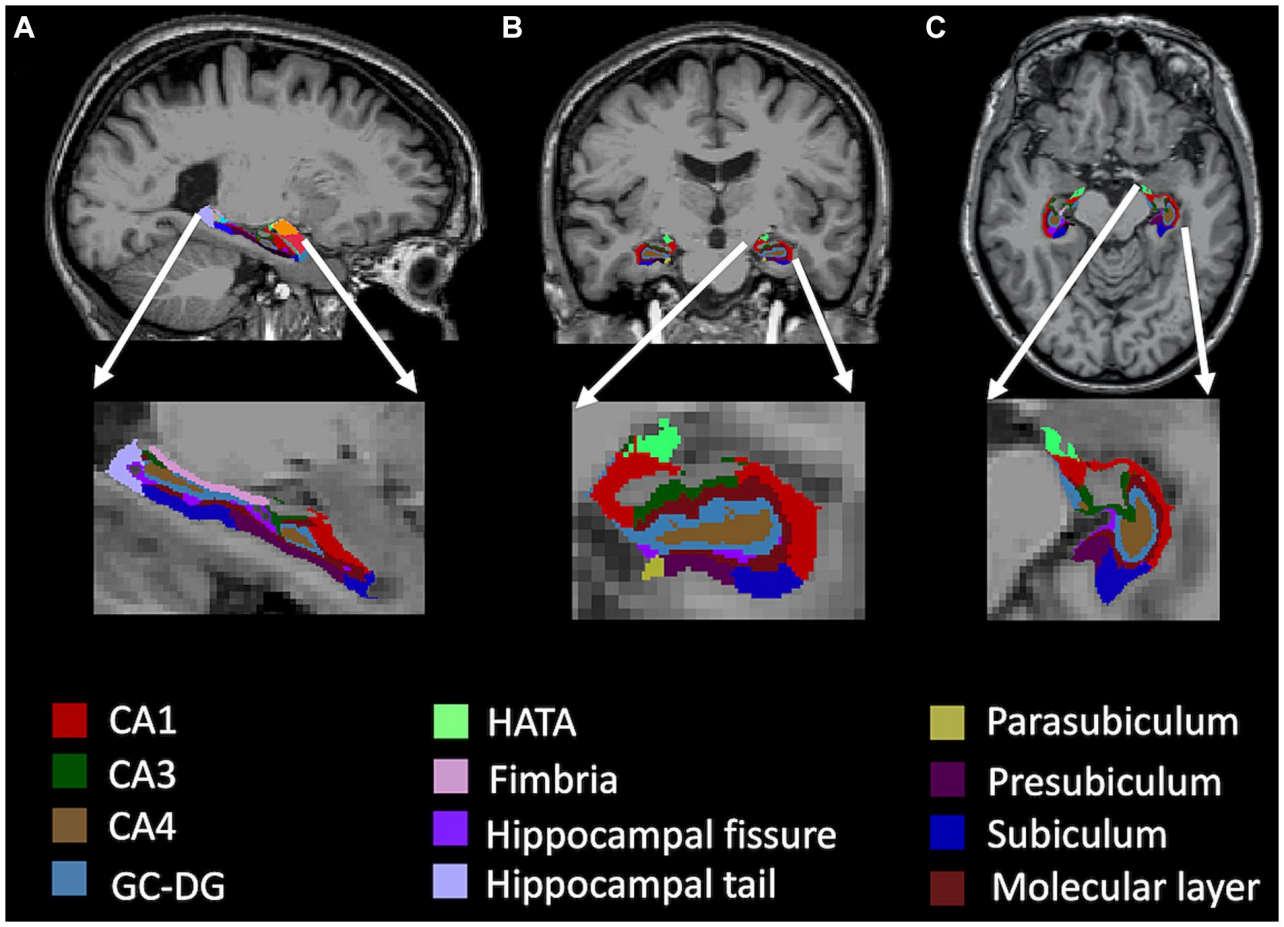
Right hippocampal subfield segmentation for one CN participant in sagittal **(A)**, coronal **(B)**, and axial **(C)** planes. GC-DG, granule cell layer of dentate gyrus; HATA, hippocampus-amygdala-transition-area.

Prior to MRI data processing, the raw MRI image of each participant was first visually inspected for artifacts and then reoriented to the standard anterior commissure (AC) and posterior commissure (PC) plane. All MRI data were processed using the FreeSurfer software suite.^4^ First, the entire hippocampal formation was segmented using the standard FreeSurfer pipeline (i.e., ‘recon-all’ command). Briefly, the MRI images were corrected for within-subject head motion. Then, non-brain tissues were removed, and the resulting images were further affine registered to the Talairach space. Subsequently, the hippocampal subfields were segmented using a Bayesian inference approach and a novel atlas algorithm of the hippocampal formations built primarily upon ultra-high resolution (~ 0.1 mm isotropic) *ex vivo* MRI data from autopsy brains (Iglesias et al., 2015). Using the atlas allowing for greater accuracy in the delineation of the boundaries within the subfields (Iglesias et al., 2015), the left and right hippocampi were segmented into twelve subfields: CA1, CA3, CA4, granule cell layer of dentate gyrus (GC-DG), hippocampus-amygdala-transition-area (HATA), parasubiculum, presubiculum, subiculum, fimbria, molecular layer, hippocampal fissure, and hippocampal tail. Figure 1 illustrates the right hippocampal subfield segmentation for one CN participant. The volume of the whole hippocampus was calculated as the sum of all hippocampal subfield volumes.

Finally, the estimated total intracranial volume (eTIV) of each subject was calculated based on the standard FreeSurfer segmentation, which was used to correct for individual differences in head size in the subsequent statistical analyses.

### Statistical analyses

#### Group differences in behavioral and clinical tests

Statistical analyses for demographics and neuropsychological data were performed with R software (version 4.1.2). Specifically, group differences among CN, MCI, and AD groups were conducted using one-way analysis of variance (ANOVA) for continuous variables (i.e., age, education, MMSE, and MoCA) and Kruskal-Wallis ANOVA for categorical variables (i.e., CDR). The Chi-squared test was used to compare the differences in gender among groups. Further, we tested the differences between CN-NC and CN-C groups in clinical and cognitive measurements (i.e., CDR, MMSE, and MoCA) using two-sample *t*-tests.

#### Comparisons of hippocampal subfield volumes between groups

We examined group differences in volumes of whole hippocampus and hippocampal subfields for the left and right hemispheres separately using regression analyses, controlling for age, gender, education, and eTIV. The results were corrected for multiple comparisons using the false discovery rate (FDR) method (*p* < 0.05). For hippocampal subfields showing significant group differences, post-hoc *t*-tests were conducted to further explore the differences between CN-NC, CN-C, MCI, and AD groups with corrections for multiple testing using the FDR method.

#### Associations between hippocampal subfield volume and delayed recall performance

Next, we examined the correlations between volume of hippocampal subfields and delayed recall scores (i.e., MIS) as measured by the MoCA in the CN-NC, CN-C, MCI, and AD groups using the regression analysis. In the regression model, MoCA MIS was the dependent variable, with volume of each hippocampal subfield as the independent variable, controlling for age, gender, education, and eTIV. Results were corrected for multiple comparisons using the FDR correction.

## Results

### Demographic and clinical data

No significant differences were observed at age or gender among CN, MCI, and AD groups. Clinical and cognitive measures (i.e., CDR, MMSE, and MoCA) showed significant differences among CN, MCI, and AD groups (*p*s < 0.001). CN-NC and CN-C groups did not differ in CDR, MMSE, or MoCA scores (*p*s > 0.05).

### Group differences in hippocampal subfield volumes

**Figure 2:**
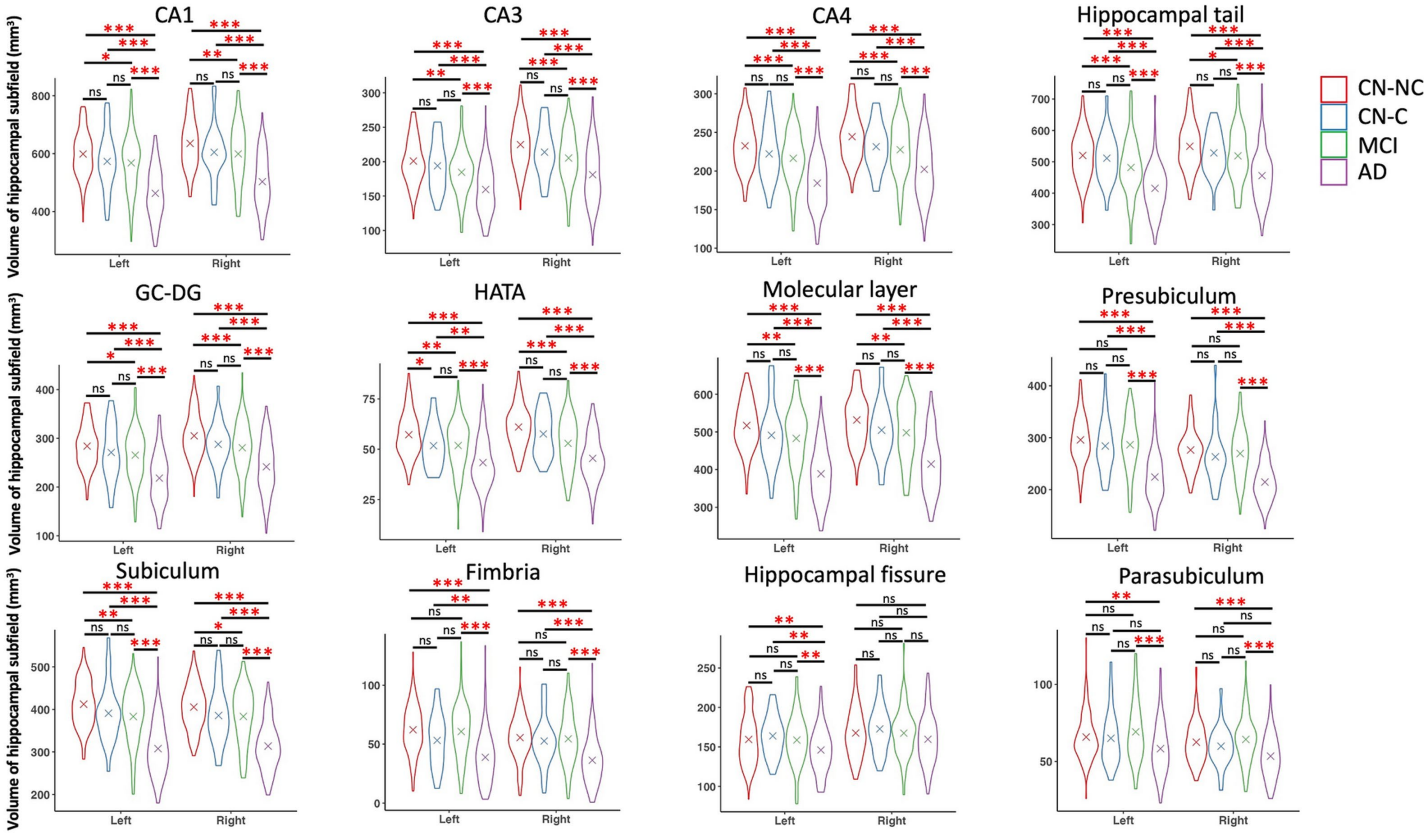
Volumes of hippocampal subfields and comparisons between CN-NC, CN-C, MCI, and AD groups. The cross indicates mean value of each group. Significant results were corrected for multiple comparisons using the FDR method. * *p* < 0.05; ** *p* < 0.01; *** *p* < 0.001; ns, not significant.

**Figure 3:**
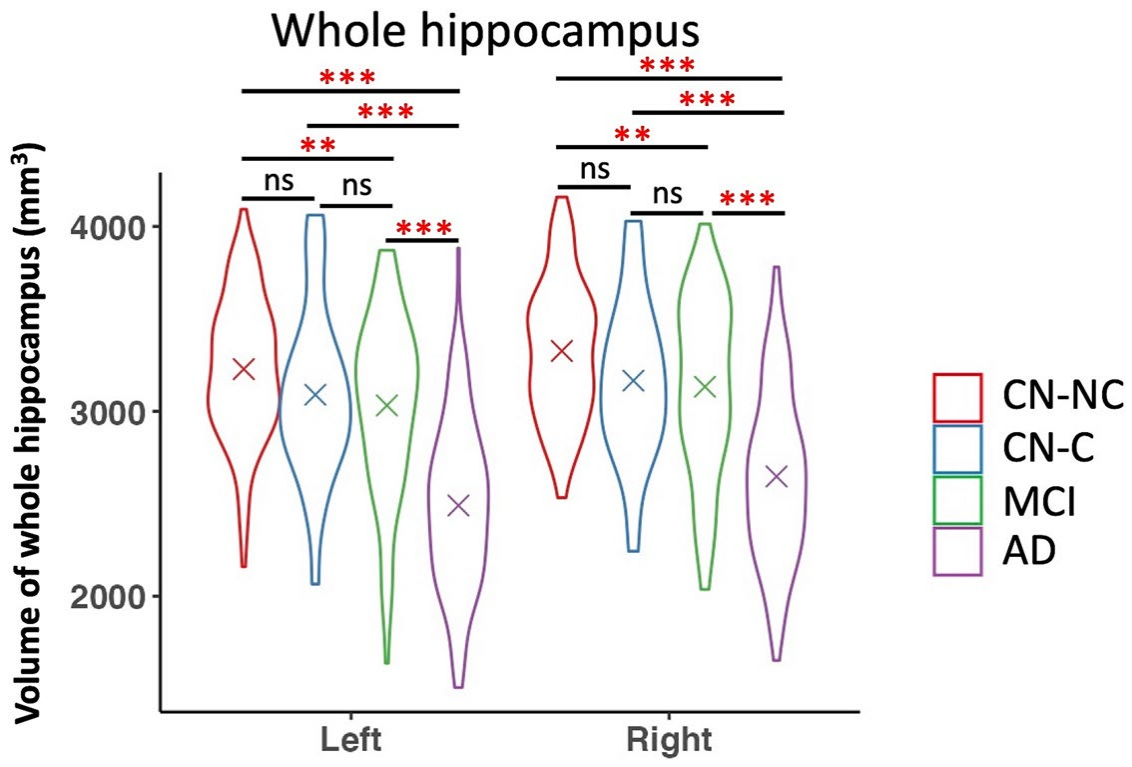
Volume of whole hippocampus and comparisons between CN-NC, CN-C, MCI, and AD groups. The cross indicates mean value of each group. Significant results were corrected for multiple comparisons using the FDR method. ** *p* < 0.01; *** *p* < 0.001; ns, not significant.

We found significant group differences in volumes of the whole hippocampus and all hippocampal subfields except for the right hippocampal fissure, controlling for age, gender, education, and eTIV; see Tables 2 and 3 for the statistical results of whole hippocampal and hippocampal subfield volumes. Figure 2 shows significant group differences in hippocampal subfield volumes between CN-NC, CN-C, MCI, and AD groups with the FDR correction. Volumes of all hippocampal subfields but bilateral presubiculum, parasubiculum, fimbria, and hippocampal fissure were significantly different between CN-NC and MCI groups. Except for the right hippocampal fissure, all other hippocampal subfields showed significant differences between CN-NC and AD, and between MCI and AD. The CN-C and AD groups differed in all hippocampal subfields but left parasubiculum and right hippocampal fissure. The significant difference between CN-NC and CN-C groups was observed in the volume of left HATA. However, no significant difference was observed between CN-C and MCI groups. Figure 3 shows significant group differences in the whole hippocampal volume between CN-NC, CN-C, MCI, and AD groups with the FDR correction.

**Table 2:** Average estimated total intracranial volume, whole hippocampal, and hippocampal subfield volumes in CN-NC, CN-C, MCI, and AD groups.

	CN-NC (*n* = 116)	CN-C (*n* = 28)	MCI (*n* = 90)	AD (*n* = 145)
	Mean ± SD	Mean ± SD	Mean ± SD	Mean ± SD
eTIV	1524.78 ± 174.86	1508.12 ± 148.74	1524.27 ± 186.51	1532.43 ± 176.84
Left whole hippocampus	3228.52 ± 375.37	3089.92 ± 460.14	3031.91 ± 473.25	2490.64 ± 446
Left CA1	598.81 ± 77	573.24 ± 99.1	567.94 ± 97.55	462.93 ± 87.21
Left CA3	200.92 ± 32.35	193.96 ± 35.3	184.6 ± 33.76	159.51 ± 35.95
Left CA4	232.54 ± 30.28	222.24 ± 35.37	216.36 ± 34.03	184.23 ± 34.79
Left subiculum	412.19 ± 52.74	390.93 ± 67.31	383.3 ± 64.87	307.77 ± 61.9
Left presubiculum	295.86 ± 43.92	284.05 ± 52.14	286.45 ± 52.4	224.29 ± 45.93
Left GC-DG	284 ± 42.48	270.96 ± 56.17	265.46 ± 50.35	218.31 ± 50.57
Left molecular layer	516.95 ± 63.51	491.16 ± 81.93	482.87 ± 78.78	388.86 ± 73.11
Left parasubiculum	65.86 ± 14.86	65.07 ± 17.37	69.22 ± 19.06	58.31 ± 16.9
Left hippocampal tail	520.06 ± 73.61	511.36 ± 74.25	481.69 ± 88.41	415.34 ± 77.97
Left HATA	57.2 ± 10.81	51.78 ± 10.62	51.85 ± 12.31	43.36 ± 12.45
Left fimbria	62.21 ± 20.29	53.21 ± 21.8	60.71 ± 22.77	38.85 ± 21.41
Left hippocampal fissure	159.43 ± 31.53	163.9 ± 25.62	158.71 ± 31.14	146.01 ± 28.53
Right whole hippocampus	3326.65 ± 369.21	3166.12 ± 428.79	3133.09 ± 472.91	2647.75 ± 450.73
Right CA1	635.34 ± 85.04	604.67 ± 91.38	599.22 ± 101.79	503.15 ± 94.47
Right CA3	224.77 ± 34.3	213.86 ± 34.92	205.22 ± 37.63	180.99 ± 41.59
Right CA4	244.5 ± 30.3	231.55 ± 29.95	227.57 ± 35.13	202.17 ± 38
Right subiculum	405.49 ± 51.1	385.55 ± 67.15	383.51 ± 64.84	313.74 ± 56.54
Right presubiculum	276.33 ± 38.23	263.18 ± 52.9	269.56 ± 47.6	214.62 ± 37.2
Right GC-DG	305.14 ± 44.71	287.66 ± 46.36	280.68 ± 52.09	241.58 ± 54.27
Right molecular layer	531.74 ± 64.29	504.61 ± 74.8	498.04 ± 79.14	414.63 ± 76.72
Right parasubiculum	62.54 ± 13.26	59.87 ± 12.91	64.31 ± 15.31	53.48 ± 14.24
Right hippocampal tail	549.19 ± 73.95	528.18 ± 68.47	518.85 ± 84.24	455.88 ± 82.69
Right HATA	61.06 ± 10.3	57.61 ± 10.95	52.91 ± 12.82	45.49 ± 11.57
Right fimbria	55.55 ± 18.87	52.68 ± 21.61	54.49 ± 22.13	36.36 ± 19.73
Right hippocampal fissure^†^	167.62 ± 30.97	172.8 ± 28.9	167.45 ± 30.43	159.63 ± 31.45

Mean and standard deviation of subfield and total hippocampal volumes in mm^3^. ^†^No significant group differences after the FDR correction.

**Table 3:** Statistical results (*p* values with the FDR correction) of whole hippocampal and hippocampal subfield volumes between CN-NC, CN-C, MCI, and AD groups.

	CN-NC vs. CN-C	CN-NC vs. MCI	CN-NC vs. AD	CN-C vs. MCI	CN-C vs. AD	MCI vs. AD
Left whole hippocampus	0.156	0.002	< 0.001	0.537	< 0.001	< 0.001
Left CA1	0.201	0.019	< 0.001	0.78	< 0.001	< 0.001
Left CA3	0.336	0.001	< 0.001	0.25	< 0.001	< 0.001
Left CA4	0.172	< 0.001	< 0.001	0.415	< 0.001	< 0.001
Left subiculum	0.115	0.001	< 0.001	0.56	< 0.001	< 0.001
Left presubiculum	0.285	0.238	< 0.001	0.815	< 0.001	< 0.001
Left GC-DG	0.244	0.01	< 0.001	0.602	< 0.001	< 0.001
Left molecular layer	0.11	0.001	< 0.001	0.597	< 0.001	< 0.001
Left parasubiculum	0.824	0.237	0.001	0.309	0.107	< 0.001
Left hippocampal tail	0.601	< 0.001	< 0.001	0.1	< 0.001	< 0.001
Left HATA	0.036	0.002	< 0.001	0.978	0.001	< 0.001
Left fimbria	0.07	0.619	< 0.001	0.128	0.003	< 0.001
Left hippocampal fissure	0.574	0.864	0.002	0.574	0.008	0.005
Right whole hippocampus	0.094	0.002	< 0.001	0.724	< 0.001	< 0.001
Right CA1	0.143	0.009	< 0.001	0.787	< 0.001	< 0.001
Right CA3	0.209	< 0.001	< 0.001	0.295	< 0.001	< 0.001
Right CA4	0.091	< 0.001	< 0.001	0.594	< 0.001	< 0.001
Right subiculum	0.123	0.011	< 0.001	0.871	< 0.001	< 0.001
Right presubiculum	0.199	0.295	< 0.001	0.478	< 0.001	< 0.001
Right GC-DG	0.121	< 0.001	< 0.001	0.523	< 0.001	< 0.001
Right molecular layer	0.097	0.002	< 0.001	0.681	< 0.001	< 0.001
Right parasubiculum	0.372	0.372	< 0.001	0.22	0.058	< 0.001
Right hippocampal tail	0.253	0.01	< 0.001	0.588	< 0.001	< 0.001
Right HATA	0.155	< 0.001	< 0.001	0.071	< 0.001	< 0.001
Right fimbria	0.71	0.71	< 0.001	0.71	< 0.001	< 0.001
Right hippocampal fissure	0.512	0.968	0.119	0.512	0.119	0.12

### Significant relationships between hippocampal subfield volumes and memory performance

**Figure 4:**
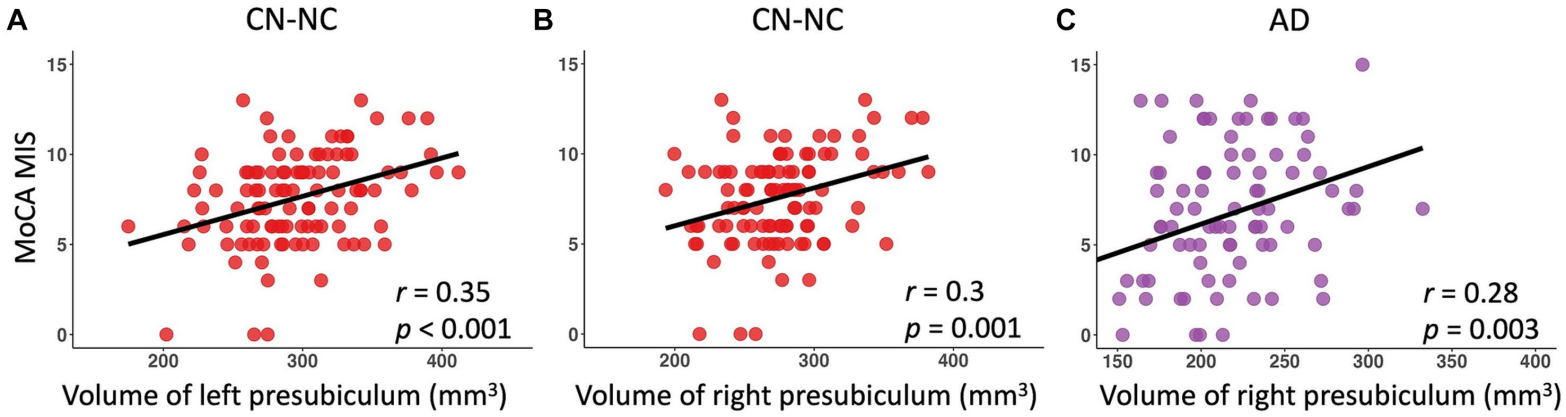
Linear regression model fitted plots between volumes of left and right presubiculum and delayed recall scores in the CN-NC group **(A,B)**, and between volume of right presubiculum and delayed recall scores in the AD group **(C)**. The *r* values are from the Pearson’s correlation analysis, and *p* values are from the regression analysis, controlling for age, gender, education, and eTIV.

There were significantly positive correlations between volumes of left and right presubiculum (left presubiculum: *p* < 0.001; right presubiculum: *p* = 0.002) and delayed recall scores (MoCA MIS) in the CN-NC group, and between volume of right presubiculum and delayed recall scores (MoCA MIS) in the AD group (*p* = 0.003), controlling for age, gender, education, and eTIV (Figure 4). The results were corrected for multiple comparisons using the FDR approach. No significant correlations were observed in other hippocampal subfields in the CN-NC or AD group, or in any hippocampal subfields in the CN-C or MCI group with multiple comparison corrections.

## Discussion

This study examined the volumetric differences in hippocampus and hippocampal subfields among CN-NC, CN-C, MCI, and AD groups. We observed significant differences between CN-NC and AD, and between MCI and AD in all hippocampal subfields but the right hippocampal fissure. The differences between the CN-C and AD groups were found in all hippocampal subfields but left parasubiculum and right hippocampal fissure. The CN-NC and MCI groups differed in most of the hippocampal subfields except for bilateral presubiculum, parasubiculum, fimbria, and hippocampal fissure. Notably, there were significant differences between CN-NC and CN-C groups in the volume of left HATA. Further, we investigated the relationships between volumes of hippocampal subfields and delayed recall scores in each group. The results showed significant associations of left and right presubiculum with delayed recall scores in the CN-NC group, and associations of right presubiculum with delayed recall scores in the AD group, but not in the CN-C or MCI group. Our data not only demonstrate the trajectories of hippocampal subfield atrophy during the pathological progression of AD, but also suggest that the atrophy of hippocampal subfields may have occurred in cognitively normal elderly individuals who later developed MCI or AD.

We presented significant atrophy of hippocampus and hippocampal subfields related to AD progression, controlling for age, gender, education, and eTIV. Though the trajectories of hippocampal subfield atrophy between CN, MCI, and AD groups have been reported in previous research (Zhao et al., 2019), here we also included a group of asymptomatic control group (i.e., CN-C) who were cognitively normal but had a change of CDR score from 0 to 0.5 for an average of 36.2 months (range: 6–105 months). Specifically, we compared the differences in volumes of hippocampal subfields between CN-NC, CN-C, MCI, and AD groups. The AD group had significantly reduced volumes as compared to CN-NC, CN-C, and MCI groups in all hippocampal subfields but the right hippocampal fissure. There were significantly reduced volumes in the MCI group as compared to the CN-NC group in bilateral CA1, CA3, CA4, subiculum, GC-DG, molecular layer, hippocampal tail, and HATA. The atrophy of these hippocampal subfields in MCI patients is consistent with that reported in previous studies (Carlesimo et al., 2015; de Flores et al., 2015; Perrotin et al., 2015; Zhao et al., 2019), suggesting different trajectories of hippocampal subfields during the progression of AD.

Contrast to our hypothesis, however, hippocampal subfield volumes did not differ between the CN-C and MCI groups. It is possible that MCI participants included in the present study had very mild cognitive decline. In fact, among 90 MCI patients, 87 had a CDR score of 0.5, which indicates questionable dementia. It is also possible that cognitive impairment had already occurred in CN-C participants although it was not detectable yet by the clinical measure used here (i.e., CDR). Nevertheless, we did find a variety of hippocampal subfield atrophy in the MCI group as compared to the CN-C participants who remained a CDR score of 0. Thus, the finding that CN-C and MCI participants had no difference in hippocampal subfield volumes may reflect atrophy of hippocampus and hippocampal subfields had already occurred in CN-C participants who had a change of CDR score from 0 to 0.5. Our finding highlights that the examination of hippocampal subfields may serve as a useful approach for early dementia detection, which could be more sensitive than clinical and behavioral measurements. On the other hand, it also suggests that the pathological processes related to AD may be present for a substantial period of time before clinical symptoms are recognized (Csernansky et al., 2005).

Here, we defined dementia converters and non-converters based on their CDR scores for an average of 36.2 months (range: 6–105 months), as CDR scores from 0 to 0.5 were used as the indicator of the onset of dementia or cognitive decline (Marquis et al., 2002; Csernansky et al., 2005). We observed significant atrophy in the volume of left HATA in CN-C as compared to CN-NC participants. The HATA, which lies in the medial region of the hippocampus and is superior to the other subfields, is supposed to be associated with information processing within the hippocampal-amygdala network and shows atrophy associated with cognitive and memory decline in normal aging (Zheng et al., 2018) and patients with Parkinson’s disease (Foo et al., 2017). While it has been reported that the atrophy of CA1 is related to the increased risk of MCI or AD (Csernansky et al., 2005; Apostolova et al., 2010; La Joie et al., 2013; Carlesimo et al., 2015) or increased risk of conversion from MCI to AD (Costafreda et al., 2011), our data did not show significant differences between CN-C and CN-NC in the volume of CA1. Future studies are needed to confirm the finding we observed here. In addition, early studies reported reduced whole hippocampal volume as predictors of cognitive decline in cognitively normal elderly participants (Marquis et al., 2002; Csernansky et al., 2005). We only observed significant volumetric differences between CN-C and CN-NC groups in the left HATA, a hippocampal subfield, instead of the whole hippocampal volume. This reflects different trajectories of hippocampal subfield atrophy, and suggests that hippocampal subfields may be better predictors than the whole hippocampus for the progression of AD.

Previous studies have demonstrated relationships of delayed recall scores with a variety of hippocampal subfields including molecular layer, GC-DG, CA3, CA4, hippocampal tail, presubiculum, CA1, subiculum, fimbria, and HATA across the adult lifespan (Zheng et al., 2018) and across CN, MCI, and AD participants (Zhao et al., 2019; Huang et al., 2020). Here, by examining the relationships in the CN-NC, CN-C, MCI, and AD group separately, we only observed significant associations of delayed recall scores with bilateral presubiculum in the CN-NC group, and with right presubiculum in the AD group, but no any associations in the CN-C or MCI group. It has been consistently reported that the atrophy of subicular complex, including presubiculum, is related to memory decline in the progression of AD (Carlesimo et al., 2015; Lindberg et al., 2017; Hartopp et al., 2019; Parker et al., 2019). Presubiculum volume loss in dementia and stroke-free older adults has been shown to be associated with cognitive decline (Evans et al., 2018). The relationships found in the CN-NC and AD groups but not in the CN-C or MCI group suggest decrease in the presubiculum volume may be markers of future memory decline in normal aging and AD. It may also explain the relationships between presubiculum atrophy and memory decline observed in both normal aging and AD across studies.

There are a few limitations in the present study. First, this study included asymptomatic control participants who later had a change of CDR score from 0 to 0.5, but lacks different types of MCI patients, such as early MCI, late MCI, and amnestic MCI. In future studies, different types of MCI patients should also be included to gain a better picture of the trajectories of hippocampal subfield atrophy related to disease progression of dementia. Second, this study was based on cross-sectional data, and the findings may be biased by the individual variabilities across groups. Thus, longitudinal samples are needed to identify early imaging markers for the transformation and prediction of Alzheimer’s disease. Third, with only cross-sectional data, the present study failed to consider the progression of AD or MCI and its correlation with the atrophy of the hippocampal formation or hippocampal subfields from a longitudinal perspective. Furthermore, we only examined the changes of hippocampus and hippocampal subfields by structural MRI data. It is supposed to provide a more comprehensive understanding of brain atrophy related to memory decline in the pathological progression of AD by including multimodal imaging data such as functional MRI, diffusion tensor imaging, electroencephalogram, and functional near-infrared spectroscopy. Finally, the hippocampal segmentation may be limited by the resolution of MRI data included in this study as some MRI data (50 out of 379) were collected from 1.5 T MRI scanners. Since ultra-high resolution (such as 5.0 or 7.0 T) MRI scanners are already available for research (Carr et al., 2017), future studies should consider running hippocampal segmentation in data collected using a 5.0 or 7.0 T MRI scanner.

## Conclusion

This study explored the changes of hippocampal subfield volumes during the progression of AD, and demonstrated that hippocampal subfield atrophy had occurred before clinical symptoms were recognized. We found significant correlations between presubiculum volume and delayed recall scores in the CN-NC and AD groups, but not in the CN-C or MCI group. Our data suggest that the volume of left HATA, a subfield supporting information processing within the hippocampal-amygdala network, may be a potential marker of the preclinical stage of AD. The relationships between presubiculum and memory performance in both CN-NC and AD groups suggest that atrophy of this subfield may be related to memory decline in both normal aging and AD. Together, these findings confirm and extend previous studies of hippocampal subfields in MCI and AD patients, and provide further insights into the hippocampal atrophy in early dementia detection.

## Data availability statement

The datasets presented in this study can be found in online repositories. The names of the repository/repositories and accession number(s) can be found at: https://github.com/Yaqiongxiao/segHA.AD.

## Ethics statement

The ethics committees/institutional review boards that approved the ADNI study are: Albany Medical Center Committee on Research Involving Human Subjects Institutional Review Board, Boston University Medical Campus and Boston Medical Center Institutional Review Board, Butler Hospital Institutional Review Board, Cleveland Clinic Institutional Review Board, Columbia University Medical Center Institutional Review Board, Duke University Health System Institutional Review Board, Emory Institutional Review Board, Georgetown University Institutional Review Board, Health Sciences Institutional Review Board, Houston Methodist Institutional Review Board, Howard University Office of Regulatory Research Compliance, Icahn School of Medicine at Mount Sinai Program for the Protection of Human Subjects, Indiana University Institutional Review Board, Institutional Review Board of Baylor College of Medicine, Jewish General Hospital Research Ethics Board, Johns Hopkins Medicine Institutional Review Board, Lifespan − Rhode Island Hospital Institutional Review Board, Mayo Clinic Institutional Review Board, Mount Sinai Medical Center Institutional Review Board, Nathan Kline Institute for Psychiatric Research & Rockland Psychiatric Center Institutional Review Board, New York University Langone Medical Center School of Medicine Institutional Review Board, Northwestern University Institutional Review Board, Oregon Health and Science University Institutional Review Board, Partners Human Research Committee Research Ethics, Board Sunnybrook Health Sciences Centre, Roper St. Francis Healthcare Institutional Review Board, Rush University Medical Center Institutional Review Board, St. Joseph’s Phoenix Institutional Review Board, Stanford Institutional Review Board, The Ohio State University Institutional Review Board, University Hospitals Cleveland Medical Center Institutional Review Board, University of Alabama Office of the IRB, University of British Columbia Research Ethics Board, University of California Davis Institutional Review Board Administration, University of California Los Angeles Office of the Human Research Protection Program, University of California San Diego Human Research Protections Program, University of California San Francisco Human Research Protection Program, University of Iowa Institutional Review Board, University of Kansas Medical Center Human Subjects Committee, University of Kentucky Medical Institutional Review Board, University of Michigan Medical School Institutional Review Board, University of Pennsylvania Institutional Review Board, University of Pittsburgh Institutional Review Board, University of Rochester Research Subjects Review Board, University of South Florida Institutional Review Board, University of Southern, California Institutional Review Board, UT Southwestern Institution Review Board, VA Long Beach Healthcare System Institutional Review Board, Vanderbilt University Medical Center Institutional Review Board, Wake Forest School of Medicine Institutional Review Board, Washington University School of Medicine Institutional Review Board, Western Institutional Review Board, Western University Health Sciences Research Ethics Board, and Yale University Institutional Review Board. The studies were conducted in accordance with the local legislation and institutional requirements. The participants provided their written informed consent to participate in this study.

## Author contributions

YX: Conceptualization, Formal analysis, Funding acquisition, Writing – original draft, Writing – review & editing. YH: Writing – review & editing. KH: Data curation, Writing – review & editing.

## Alzheimer’s Disease Neuroimaging Initiative

Data used in preparation of this article were obtained from the Alzheimer’s Disease Neuroimaging Initiative (ADNI) database (adni.loni.usc.edu). As such, the investigators within the ADNI contributed to the design and implementation of ADNI and/or provided data but did not participate in analysis or writing of this report. A complete listing of ADNI investigators can be found at: http://adni.loni.usc.edu/wp-content/uploads/how_to_apply/ADNI_Acknowledgement_List.pdf.

## Footnotes

^1^ https://adni.loni.usc.edu/

^2^ www.adni-info.org

^3^ www.loni.ucla.edu/ADNI

^4^Version 7.2.0, https://surfer.nmr.mgh.harvard.edu/.
